# The Water Supply of Buffalo

**Published:** 1907-02

**Authors:** 


**A Monthly Review of Medicine and Surgery.**

*EDITOR:*

WILLIAM WARREN POTTER, M. D.

All communications, whether of a literary or business nature, books for review and exchanges, should be addressed to the editor 238 Delaware Ave., Buffalo, N. Y.

Vol. LXII. FEBRUARY, 1907. No. 7

### The Water Supply of Buffalo.

THE question of a pure water supply for Buffalo is again being agitated. The people of the city, if they have anything to say about the matter at all, say it in the privacy of their own homes when they see the muddy water which is their portion after every little wind storm, and they say what they have to say where it can not affect their standing as christians and law-abiding people. It is difficult to understand just why Buffalo, situated as it is, at the very threshold of unlimited supply, should be so illy-provided with so necessary an article as pure water. Whether the fault lies with the people themselves or with the lobbyists and the grafters who have delayed matters for their own good fortune, or with the ingrown Buffalo tendency to wait in the hope that the water problem may work out its own salvation, The Journal does not pretend to say. But with the experience of scores of other cities and countless little towns which have provided their peoples with decent water, it surely should not be a difficult matter to form some comprehensive plan by which something tangible might be evolved.

There has been water talk enough to saturate a dozen cities of the size of Buffalo; all it has resulted in has been the employment of experts to advise regarding the system; the frequent and suspiciously persistent shriek of warning to boil the water, a cry which is so commonplace that it has become a joke and taken its place among the standing jibes of the local newspaper humorists.

There has been argument for and against this system and that; to build an intake far out in the lake would be best; to build such an intake would be the height of folly and idiocy; a filtration plant is what is needed; filtration is a curse and a fraud; a reservoir in the Hamburg hills is the city's only salvation; such a work would wreck the city and the supply would be inadequate; and so it goes, with no result in sight and no relief.

The latest effort to get something like a human idea of what the situation really is—the consideration of the water question in its broadest sense by the Academy of Medicine at a recent meeting,—was productive of nothing at all except a triple row of damnations from Col. Ward, the Commissioner of Public Works. Of course, physicians do not know a thing about the water supply. Admitting that scientific men who make a business of proper water supply are invaluable as advisors in such cases, it still occurs to the casual observer that it might be of benefit to the city if the mayor should select several professional gentlemen who have neither axes to grind nor jobs to hunt, and get their views in official form, not alone on the present needs of the city in relation to water, but on the best method of supplying the necessity in the future. Let these men be so well placed that they would not be amenable to the flatteries of the filtration plant or other water contractors; their opinion would have the advantage of being based on what a good water supply should be for the people and not how much there is in the construction of a plant or an intake.

St. Louis, Boston, Washington and other cities have been through the same trouble; the only difference is that these cities have met the difficulty promptly—whether adequately or not, need not be here considered at this time. The fact that they, together with Cleveland and Detroit, have done something, means much for their progressiveness; this fact alone may be one of the reasons why both Cleveland and Detroit have dropped Buffalo behind them in civic progress and census returns.

On the surface Buffalo appears to be suffering at the present time from business anemia and a certain congenital sluggishness.

The Academy of Medicine made a good beginning in taking up the water question. Its mistake was in inviting the office-holder and opening the way for the Aldermanic farce of "hearing interested parties." What the Academy of Medicine should do is to wade into civic conditions affecting the public health, and wade deep; make of it as thorough a job as maybe; probe deep; praise with unstinted praise where good is found; where bad is found, damn loudly with unstinted damnation—and keep it up.

Buffalo has pure water within reach. The only thing necessary is to get it, and get it by a method which will insure not only purity but a quantity sufficient for the needs of the city of to-day and the inland metropolis of the future.

What Buffalo needs is civic surgery.

At present there is every probability that the proposed new intake to the Emerald channel will be three years building. 'Till then, Buffalo will suffer for water.

